# Early mortality in *STXBP1-*related disorders

**DOI:** 10.1007/s10072-024-07783-3

**Published:** 2024-10-11

**Authors:** Francesca Furia, Charlene Son Rigby, Ingrid E. Scheffer, Nicholas Allen, Kate Baker, Christian Hengsbach, Josua Kegele, James Goss, Kathleen Gorman, Misra-Isrie Mala, Francesco Nicita, Talia Allan, Alberto Spalice, Yvonne Weber, European STXBP1 consortium (ESCO), Ganna Balagura, Bruria Benzeev, Hilgo Bruining, Alejandra Darling, Francesca Furia, Ángeles García Cazorla, Misra-Isrie Mala, Mathieu Milh, Rikke Steensbjerre Møller, Hannah Stamberger, Pasquale Striano, Steffen Syrbe, Kim Marie Thalwitzer, Matthijs Verhage, Sarah Weckhuysen, STXBP1 foundation, Guido Rubboli, Rikke S. Møller, Elena Gardella

**Affiliations:** 1 Department of Epilepsy Genetics and Personalized Treatment, Danish Epilepsy Center, Member of the European Reference Network EpiCARE, University of Southern Denmark, Dianalund, Denmark; 2 Institute for Regional Health Services, University of Southern Denmark, Odense, Denmark; 3 STXBP1 Foundation, Holly Springs, USA; 4 Epilepsy Research Centre, Department of Medicine, University of Melbourne, Austin Health, Victoria, Australia; 5 Department of Paediatrics, University of Melbourne, Royal Children’s Hospital, Florey Institute and Murdoch Children’s Research Institute, Melbourne, VIC Australia; 6 Department of Paediatrics, University of Galway, Galway, Ireland; 7 MRC Cognition and Brain Sciences Unit, University of Cambridge, Cambridge, U.K.; 8 Department of Neurology and Epileptology, Hertie Institute of Clinical Brain Research, University of Tubingen, Tubingen, Germany; 9 Department of Neurology and Clinical Neurophysiology, Children’s Health Ireland at Temple Street, Dublin, Ireland; 10 School of Medicine and Medical Science, University College Dublin, Dublin, Ireland; 11 Department of Human Genetics, Clinical Genetics Section, Amsterdam University Medical Center, Amsterdam, the Netherlands; 12 Functional Genomics, Department of Human Genetics, Center for Neurogenomics and Cognitive Research, Amsterdam UMC, Amsterdam, Netherlands; 13 Unit of Neuromuscolar and Neurodegenerative Disorders, IRCCS Bambino Gesù Children’s Hospital, Rome, Italy; 14 Department of Maternal Sciences, Pediatric Division, Sapienza University, Rome, Italy; 15 Department of Epileptology, Neurology, University RWTH Aachen, Aachen, Germany; 16 European STXBP1 Consortium, Amsterdam, The Netherlands; 17 Department of Neurology, Danish Epilepsy Center, Member of the European Reference Network EpiCARE, Dianalund, Denmark; 18 Institute of Clinical Medicine, University of Copenhagen, Copenhagen, Denmark; 19 Department of Neurophysiology, Danish Epilepsy Center, Member of the European Reference Network EpiCARE, Dianalund, Denmark

**Keywords:** *STXBP1*, Developmental and Epileptic Encephalopathy (DEE), Early mortality, Sudden unexpected death in epilepsy (SUDEP)

## Abstract

**Introduction:**

Pathogenic variants in *STXBP1* cause a spectrum of disorders mainly consisting of developmental and epileptic encephalopathy (DEE), often featuring drug-resistant epilepsy. An increased mortality risk occurs in individuals with drug-resistant epilepsy and DEE, with sudden unexpected death in epilepsy (SUDEP) often the major cause of death. This study aimed to identify the rate and causes of mortality in *STXBP1*-related disorders.

**Methods:**

Through an international call, we analyzed data on individuals with *STXBP1* pathogenic variants, who passed away from causes related to their disease.

**Results:**

We estimated a mortality rate of 3.2% (31/966), based on the *STXBP1* Foundation and the *STXBP1* Global Connect registry. In total, we analyzed data on 40 individuals (23 males) harboring pathogenic *STXBP1* variants, collected from different centers worldwide. They died at a median age of 13 years (range: 11 months—46 years). The most common cause of death was SUDEP (36%), followed by pulmonary infections and respiratory complications (33%). The incidence of SUDEP peaked in mid-childhood, while non-SUDEP causes were more frequent in early childhood or adulthood (p = 0.006). In the most severe phenotypes, death was related to non-SUDEP causes (*p* = 0.018).

**Conclusion:**

We found a mortality rate in *STXBP1*-related disorders similar to other DEEs, with an early age at death and SUDEP as well as pulmonary infections as the main cause of death. These findings assist in prognostic evaluation and genetic counseling for the families. They help to define the mortality risk of *STXBP1*-related disorders and implement preventative strategies.

**Supplementary Information:**

The online version contains supplementary material available at 10.1007/s10072-024-07783-3.

## Introduction

The gene *STXBP1* encodes for the syntaxin-binding protein 1 (STXBP1) that interacts with the soluble NSF attachment protein receptors (SNARE) leading to synaptic vesicles docking and fusion, allowing the release of neurotransmitters at all synapses [1].

Pathogenic variants in *STXBP1* cause a range of developmental and epileptic encephalopathies (DEEs) [2–4]. *STXBP1*-related DEEs include Early Infantile DEE, Infantile Epileptic Spasms syndrome and Dravet syndrome [5]. Tonic seizures and epileptic spasms are the main seizure types, and are often drug-resistant, with periods of spontaneous worsening [4].

Studies have showed an increased risk of mortality in individuals with epilepsy [6], mainly due to sudden unexpected death in epilepsy (SUDEP), with an incidence of SUDEP of 0.35/1000 person-years, which increases in patients with chronic (1–2/1000) and drug-resistant (3–9/1000) epilepsy [7].

Recent studies have suggested an elevated risk of mortality in individuals with DEE, primarily due to sudden unexpected death in epilepsy (SUDEP) and other complications. The mortality risk in individuals with DEEs is further increased by their susceptibility to respiratory comorbidities including infection [8, 9]. There is a paucity of studies for the monogenic DEEs and new etiology-specific syndromes, documenting mortality outcome measures, and longitudinal (natural history) studies, in general are severely lacking. This study aims to elucidate the rate and specific causes of mortality in a cohort of individuals with *STXBP1*-related disorders, contributing to better clinical management and family counseling.

## Methods

We performed a literature review to collect data on mortality on all reported individuals with *STXBP1*-related disorders by performing a PubMed search (last search: January 2024) with the term *STXBP1*. We selected 229 clinical papers, and contacted the corresponding authors to ask (a) for additional information about death circumstances and (b) death since publication.

In addition, we collected unpublished cases from the *STXBP1* Foundation and the European *STXBP1* Consortium (ESCO). Information about the number and ages of individuals (alive and deceased) with *STXBP1* pathogenic variants included in the international registry of the *STXBP1* Foundation and the *STXBP1* Global Connect consortium were obtained. All individuals included in this study carried a *STXBP1* pathogenic variants. However, due to single countries legal rules, for some of them it was not possible to get back to the original charts and collect details about the variant type and position, since the records after death were not be covered by the US HIPAA law any more.

In order to understand the circumstances surrounding each individual’s death, their treating physicians completed a spreadsheet and we conducted a semi-structured interview of the close relatives of each individual. Data included demographic and genetic information, epilepsy features, cognitive and motor development, comorbidities, and death circumstances (age, cause, neurological and clinical conditions at death and in three months prior to death).

SUDEP was defined and classified according to Nashef criteria [10]. Considering that all individuals had ongoing seizures, we classified the *STXBP1-*related disorders phenotype as: 1) profound, when the individuals had severe intellectual disability (ID) and were not-ambulatory, 2) severe, when they had severe ID but were able to walk, and 3) moderate, when they had mild/moderate ID and were able to walk autonomously. Only two individuals, having had seizures for a brief period before death, have been classified as developmental encephalopathy (DE) + seizures, with a moderate phenotype.

Descriptive statistics were used to determine the rate of death and its relationship with phenotypic and genotypic variables (t-test and chi-square test). Written informed consent was obtained from all families participating in this study.

## Results

We studied 40 individuals (23 males) with *STXBP1*-related disorders who died at a median age of 13 years (range: 11 months to 46 years), including 10 previously published cases [2, 4, 11–14] and 30 novel cases.

**Fig. 1:**
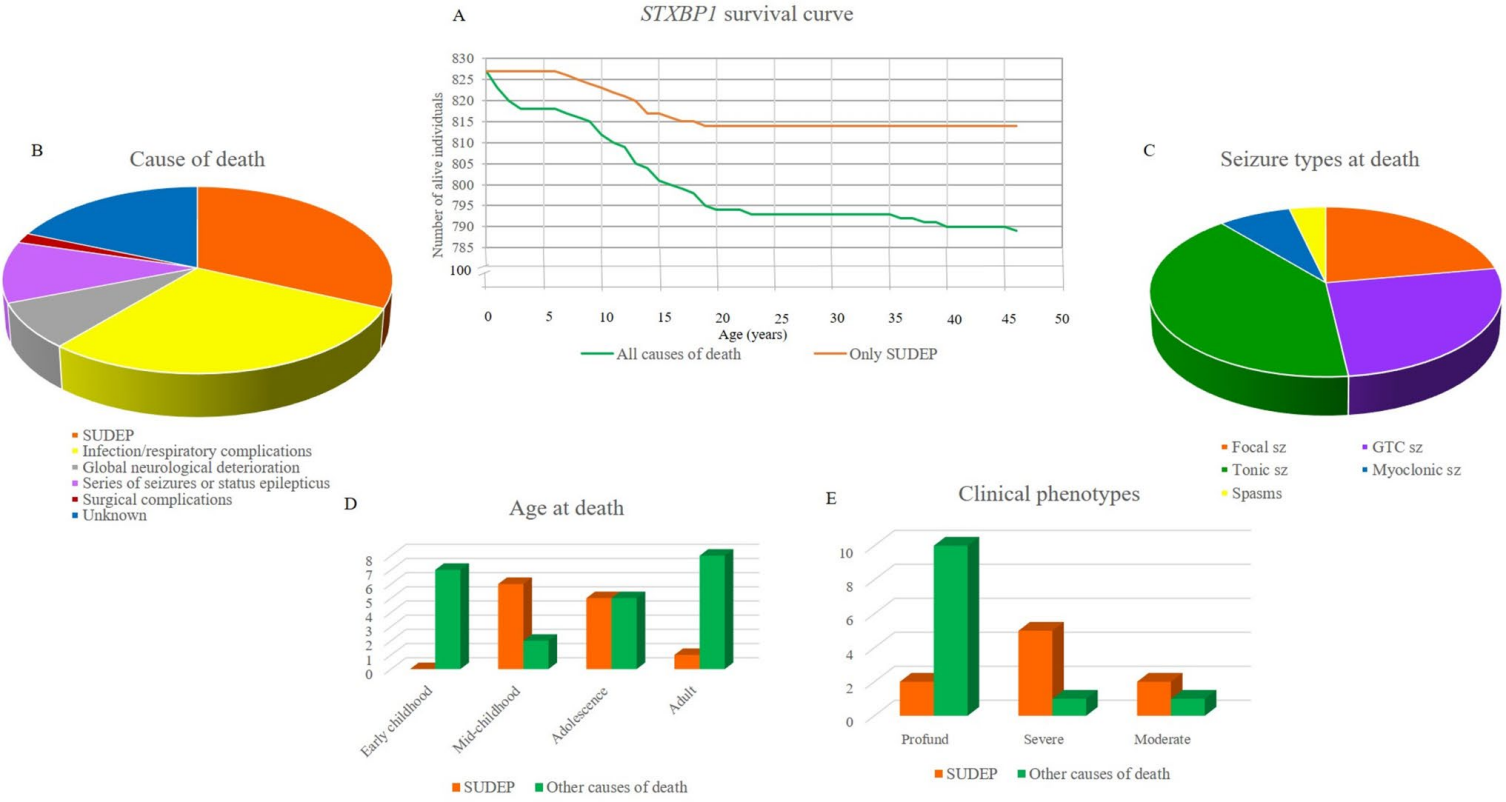
Survival curve and clinical features at death of 40 individuals deceased with ***STXBP1***-related disorders. **A** The survival curve shows the number of individuals alive at different ages from the international *STXBP1* Foundation registry. **B** The main cause of death in our cohort is represented by SUDEP, followed by infections mainly pulmonary and respiratory complications, series of seizures / status epilepticus, global neurological deterioration, surgical complications. **C** In our cohort SUDEP occurs more often in mid-childhood and adolescence and non-SUDEP causes of death in early childhood and adulthood. **D** SUDEP is reported more often in severe phenotypes, and other causes of death in profound phenotypes

The *STXBP1* database of the *STXBP1* Foundation and the *STXBP1* Global Connect included 966 individuals. We estimated a *STXBP1*-mortality rate of 3.2% (31/966 individuals) with an incidence of around 3/1000 person-year (Fig. 1A). We collected details about nine more deceased individuals from other European collaborating centers.

The cause of death was known for 33/40 individuals; detailed clinical data were available for 22 individuals and detailed genetic information for 21 (Table 1 and supplementary material). Denominators in the results depended on the number of cases for whom the details were available.

**Table 1:** Mortality and clinical features of 40 individuals with *STXBP1*-related disorders

ID	Published/ Unpublished	*STXBP1* variant (*)	Sex	Age at sz onset	Age at death	Cause of death	Phenotype (syndrome)	Latest sz types	Response to ASM
1	Unpublished	p.(Arg406Gly)	f	Birth	11 mo	Respiratory infection	Profound DEE	T, My	Refractory
2	Stamberger et al., 2016 (pt. 5) [2]	p.Gln359*	m	1 d	1 y	Respiratory infection	Profound DEE (WS)	NA	Refractory
3	Balagura et al., 2022 (pt.32) [11]	p.(Glu487Asp)	m	17 d	1 y	Respiratory complications	Profound DEE	NA	NA
4	Unpublished	Position NA	m	NA	1 y	Unknown^(^**^)^	NA	NA	NA
5	Allen et al., 2016 (pt.4) [12]	p.(Pro79Leu)	f	5 d	2 y	Unknown ^(^**^)^	Profound DEE (OS)	T, FIAS	NA
6	Nicita et al., 2015 (pt.4) [13]	9q33.3 → 9q34.12 del, about 4 Mb	f	5 wks	2 y	Cardiopulmonary arrest	Profound DEE (DLS)	F, My	Refractory
7	Balagura et al., 2022 (pt.5)	p.(Gln359*)	m	3 d	2 y	Respiratory complications	Profound DEE	NA	NA
8	Unpublished	Position NA	m	NA	3 y	Subsequent seizures	NA	NA	NA
9	Unpublished	Position NA	m	NA	3 y	Heart failure due to respiratory infection	NA	NA	NA
10	Unpublished	p.(Arg190Gln)	m	6 mo	7 y	Definite SUDEP	Moderate DEE	GTC	Refractory
11	Unpublished	p.(Cys44Tyr)	m	3 mo	8 y	Probable SUDEP	Moderate DEE	F, F- > GTC	Ongoing sz
12	Unpublished	Position NA	m	NA	9 y	Definite SUDEP	NA	NA	NA
13	Unpublished	Position NA	m	NA	10 y	Respiratory infection	NA	NA	NA
14	Unpublished	Position NA	NA	NA	10 y	Unknown^(^**^)^	NA	NA	NA
15	Unpublished	p.(Arg292Cys)	f	18 mo	10 y	Definite SUDEP	Profound DEE	GTC	Refractory
16	Carvill et al., 2014 (pt.T1915) [14]	p.(Glu283Lys)	m	11 mo	11 y	Probable SUDEP	Severe DEE (DLS)	T	Refractory
17	Balagura et al., 2022 (pt.17) [11]	p.(Ile19_Lys20delinsMet)	m	17 mo	11 y	Refractory SE	Moderate DEE	NA	NA
18	Stamberger et al., 2016 (pt.10) [2]	p.(Arg551Cys)	m	18 mo	12 y	Possible SUDEP	Severe DEE (EOEE)	NA	Refractory
19	Unpublished	Large deletion of 52 genes including *STXBP1*	f	3 mo	13 y	Possible SUDEP	Severe DE + S	GTC	Responsive
20	Unpublished	del(9) (q34.11), dup(19) (q13.32)	m	1 wk	13 y	Subsequent seizures	Profound DEE	NA	Refractory
21	Unpublished	Position NA	NA	NA	13 y	Complication after surgery	NA	NA	NA
22	Unpublished	Position NA	f	NA	13 y	Respiratory infection	NA	NA	NA
23	Unpublished	p.(Leu130fs)	m	2 wks	14 y	Global clinical compromise	Profound DEE	GTC	Refractory
24	Unpublished	p.(Arg551Cys)	m	NA	15 y	Possible SUDEP	Profound DEE	F, GTC	Refractory
25	Unpublished	Position NA	m	NA	15 y	Definite SUDEP	NA	NA	NA
26	Unpublished	Position NA	f	NA	15 y	Definite SUDEP	NA	NA	NA
27	Unpublished	p.(Arg388*)	f	15 y	16 y	Possible SUDEP	Severe DE + S	T	Sz onset 3 wks before death
28	Unpublished	Position NA	m	NA	17 y	Unknown (not SUDEP)^(^**^)^	NA	NA	NA
29	Unpublished	Deletion 9q44	f	Birth	18 y	Global clinical compromise	Profound DEE	F	Refractory
30	Unpublished	p.(Arg551Cys)	f	9 mo	19 y	Possible SUDEP	Severe DEE	F, GTC	Refractory
31	Unpublished	Position NA	f	5 d	19 y	Global clinical compromise	Profound DEE	GTC, Ab	Refractory
32	Unpublished	Position NA	m	NA	19 y	Unknown^(^**^)^	NA	NA	NA
33	Unpublished	Position NA	f	NA	20 y	Covid infection	NA	NA	NA
34	Stamberger et al., 2022 (pt.4)	p.(Ser121Ilefs*21)	m	1 d	23 y	Sepsis	Profound DEE (OS)	NA	Refractory
35	Stamberger et al., 2022 (pt.3)	p.(Asp234Gly)	m	10 mo	36 y	Complication after surgery (nfs)	Severe DEE (EOEE)	NA	Refractory
36	Unpublished	Position NA	m	NA	38 y	Chocking after a seizure	NA	NA	NA
37	Unpublished	Position NA	f	NA	40 y	Sepsis	NA	NA	NA
38	Unpublished	Position NA	f	NA	46 y	Respiratory infection	NA	NA	NA
39	Unpublished	Position NA	m	NA	NA	Unknown^(^**^)^	NA	NA	NA
40	Unpublished	Position NA	f	NA	NA	Unknown^(^**^)^	NA	NA	NA

(*) All individuals included in this study carried a *STXBP1* pathogenic variants. Due to different single countries legal rules, for some of them it was not possible to get back to details about the variant type and position

(**) All individuals passed away for causes directly or indirectly related to their disease

*Ab*, absences, *d *, day(s), *DLS*, Dravet-like syndrome, *EOEE*, early onset epileptic encephalopathy, *DEE*, developmental epileptic encephalopathies, *DE + S*, developmental encephalopathy + seizures, *F,* focal, *FIAS*, focal impaired awareness, *f*, female, *GTC*,  generalized tonic clonic, *m*, male, *mo*, months, *my*, myoclonic, *NA*, not available, *nfs*, not further specified, *OS*, Ohtahara syndrome, *pt*, patient, *sz*, seizure, *SUDEP*, sudden unexpected death in epilepsy, *T*, tonic, *wks*, weeks, *y*, years, *WS*,West syndrome

### Causes of death

SUDEP was reported in 12/33 individuals (36%), and was classified as definite in 5, probable in 2, and possible in 5. Other causes of death were: infections, mainly pulmonary (*n* = 6) and respiratory complications (11/33, 33%), series of seizures or status epilepticus (defined as a seizure lasting at least 30 min) (4/33, 12%), global neurological deterioration (3/33, 9%), complications of surgery (2/33, 6%), and cardiopulmonary arrest (1/33, 3%). In 7/33 (21%) individuals, the cause of death was unknown (Fig. 1B).

The median age of SUDEP was 12 years (range: 7–19 years), with peaks during mid-childhood (6 to 12 years; 6/12, 50%) and adolescence (13 to 19 years; 5/12, 42%). The age at death for non-SUDEP causes was more variable (p = 0.006) with a median age of 13 years (range: 11 months—46 years). Peaks occurred during early childhood (2 to 6 years; 7/22, 32%) and during the second to third decade of life (7/22, 36%) (Fig. 2D).

All causes of death were observed in all phenotypic groups. SUDEP was more frequent in the “severe” group (5/9, 55.5%), while non-SUDEP causes occurred mainly in the “profound” group (10/12, 83%) (p = 0.018) (Fig. 2E). The three individuals who died in the context of a global neurological deterioration had profound phenotypes.

### Clinical presentation in the three months before death

In 11/33 (33%) individuals, an infection, typically affecting the respiratory tract (*n* = 10), was reported. In four individuals increased seizure frequency occurred; refractory status epilepticus was reported as the cause of death in one individual, and subsequent seizures in two. Hospitalization for seizures in the three months before death was necessary for two individuals: one for seizure recurrence after 12 years of seizure-freedom, and one for seizure onset three weeks before his death. All individuals but one, were on treatment with 1–2 (50%) or ≥ 3 (50%) anti-seizure medication (ASM). No reduction in ASM or poor compliance were reported.

### Clinical background

None of the individuals, for which clinical and genetic data were available, reported a family history of sudden death. All presented with ID that was severe in 18/22 (82%); in four, developmental regression was observed (in all cases during childhood; one presented a second period of regression at puberty). The majority of individuals was non-verbal (15/19, 79%), 12/21 (57%) were non-ambulatory, 2/21 (9.5%) walked with support, and 7/21 (33%) walked autonomously.

All 40 individuals had epilepsy, with a median age at onset of 2 weeks (range: birth – 15 years), and all but three had drug-resistant epilepsy. A detailed seizure description was available for 14 individuals; the most common seizure types were generalized tonic–clonic (GTC) (7/14, 50%), focal (7/14, 50%), tonic (4/14, 29%), and myoclonic seizures (2/14, 14%) (Fig. 2C) at follow up near to prior SUDEP outcome. Seizure duration ranged from a few seconds to 5 min, and frequency from multiple/day to multiple/month. Other common neurological features were ataxia/postural instability (7/12 able to stand/walk, 58%), hypertonia (8/20, 40%), hypotonia (7/20, 35%), tremor (4/20, 20%), dystonia (3/20, 15%), fine motor/coordination impairment (3/20, 15%), neurogenic bladder (3/20, 15%), cortical visual impairment (3/20, 15%), and microcephaly (2/20, 10%).

We classified 13/22 (59%) individuals as “profound”, 6/22 (27%) as “severe”, and 3/22 (14%) as “moderate” phenotype. Brain magnetic resonance imaging (MRI) was performed in 11 individuals revealing non-specific changes, such as cerebral atrophy, subcortical hyper-intensities and corpus callosum hypoplasia.

The 21 individuals with available genetic information harbored 19 *STXBP1* variants, including 10 (53%) missense and nine (47%) null variants. All variants were de novo, except one inherited from an unaffected father (mosaic 15%). A recurrent missense variant [c.1651C > T, p.(Arg551Cys)], was found in three unrelated individuals with “severe” phenotype and drug-resistant epilepsy with ongoing GTCs, all died of possible SUDEP.

The rate of deceased individuals with missense variants was 12/21 (57%), and with null variants was 9/21 (43%). SUDEP was the cause of death in 7/11 (64%) individuals with missense variants (for one the cause was unknown), and in 2/9 (22%) with null variants. In both groups, most individuals had a very severe/severe phenotype (88% missense versus 89% truncating), severe ID (84% versus 87.5%), and high seizure burden (multiple seizures/day in 50% versus 67%). In both groups, the most common seizure types were GTC (62.5% missense versus 40% truncating), tonic (37.5% versus 20%), and focal onset seizures (37.5% versus 40%). Most individuals were not-ambulatory and not-verbal, with a higher but not statistically significant percentage in the truncating group (78% and 89%) compared to missense (55% and 67%).

## Discussion

We defined the mortality rate in a large cohort of 40 individuals with *STXBP1*-related disorders and described their circumstances of death.

The estimated incidence of mortality in our cohort was around 3/1000 person-year, considerably higher than the incidence in the general epilepsy population (1.2–1.3/1000 person-year) [16]. The mortality rate was 3.2%, similar to that observed in other DEEs, such as *SCN8A*-DEE (5%) [9], and other genetic DEEs (6%) and lower than Dravet syndrome (8.6%) [17].

SUDEP is the most common cause of death in in both, children (19%) and adults (24%), with epilepsy of any etiology [18, 19]. SUDEP was responsible for 36% of the mortality in our *STXBP1* cohort, similarly to that observed in other monogenic DEEs, such as *SCN8A*-DEE (30%) [9], and *SCN2A*-DEE (33%) [17], but lower than in Dravet syndrome (52%) [17]. The pathophysiological mechanisms behind SUDEP remain unknown. Several hypotheses have been made, most involving cardiac, respiratory or cerebral dysfunction, either isolated or in combination [15], with reported cases, in humans and murine models, of GTC seizures followed by severe respiratory depression/arrest and/or prolonged bradycardia leading to death [20–23]. Interestingly, in one of our individuals who died from SUDEP, ictal hypoxia was reported, and, in another, suffocation after a GTC seizure was described. No significant cardiac or respiratory abnormalities were reported in the rest of our cohort.

The remaining 64% of cases of death in our *STXBP1* cohort were related to non- SUDEP causes, including infections, mainly pulmonary, in neurologically compromised individuals (28%), or series of seizures/status epilepticus (12%). The age distribution of different causes of death, with SUDEP occurring more frequently in mid-childhood (median age: 12 years), and non-SUDEP causes being more prominent in early childhood (median age: 2 years) or adulthood (median age: 23 years), mirrors what has been observed in *SCN8A*-DEEs [24]. Individuals with the most severe phenotypes with profound impairment were more likely to die of non-SUDEP causes, as previously observed in *SCN8A*-DEEs [9] and other monogenic DEEs [17].

An appropriate preventive approach to early death in *STXBP1*-related disorders requires careful treatment and monitoring of epilepsies (optimization of ASM, reduction of the frequency of GTC seizures, limitation of seizure triggers such as sleep deprivation and illness), and adequate education of caregivers (optimization of adherence to treatment, assessment of risk awareness). A detailed risk quantification is of pivotal importance and can be obtained through regular specialistic follow-up and safety checklists and SUDEP scores. Prevention strategies for SUDEP also include nocturnal supervision, listening devices, and seizure detection devices. Since sleep and prone position are vulnerability factors for SUDEP, nocturnal supervision and safe positioning during sleep are considered the cornerstone of prevention. Seizure detection devices may help alert caregivers so that appropriate intervention and stimulation can be provided after a seizure [25]. The identification and treatment of all the clinical situations that can further increase the fragility of the individuals with *STXBP1-*related disorders, should also be identified and managed to reduce the non-SUDEP causes of death.

For the individuals with available genetic data, we did not find a correlation between different causes of death and particular *STXBP1* variant positions. Only one variant [c.1651C > T, p.(Arg551Cys)] was found in three individuals, but it is known from the literature that it has a high percentage of recurrence [26]. The lack of a clear genotype–phenotype correlation is in line with a study exploring epilepsy and developmental trajectories in a large cohort of individuals with *STXBP1* variants [26]. Regarding the variant type, missense and null variants were equally represented in our cohort, with a higher percentage of SUDEP in the missense group compared to the null group, despite their similar phenotype. However, the limited number of patients does not allow us to come to definitive conclusions. Further studies on larger cohorts will help to better define this point.

## Conclusion

We provide an estimate of the mortality rate and a description of the mortality circumstances in *STXBP1*-related disorders. Thanks to a larger cohort compared to previous studies, we had the possibility to characterize death circumstances in individuals with *STXBP1*-related disorders. We found an early median age of death, and SUDEP as well as pulmonary infections as the main causes of death. These results are important for family and prognostic counselling and highlight the need for a vigilance and prevention in individuals with *STXBP1*-related disorders.

## Supplementary Information

Below is the link to the electronic supplementary material.

Supplementary file1 (DOCX 23 KB)

## Data Availability

The data that support the findings of this study are available, upon request, from the corresponding author within a reasonable time after the publication of this article. With possible restrictions due to privacy or ethical concerns.
