# Supplementary material for: Early mortality in STXBP1-related disorders
Source: Neurol Sci. 2024 Oct 11;46(3):1339–47. doi: 10.1007/s10072-024-07783-3 (PMC11828786; doi:10.1007/s10072-024-07783-3)
Supplement: Supplementary file 1 — Supplementary file1 (DOCX 23 KB) [file 10072_2024_7783_MOESM1_ESM.docx]

**Supplementary material**

Clinical summaries of 15 individuals, for which detailed clinical information was available.

**Proband 1**

Proband 1 was a girl, with a paternally inherited missense variant c.1216C>G, p.(Arg406Gly), deceased at 11 months of life for pneumonia. Family history was negative for sudden death or epilepsy. She had global developmental delay (she did not gain any skills), epilepsy, hypertonia, cortical visual impairment and excessive sleepiness. Seizures started at birth with GTC seizures. Tonic and myoclonic seizures were described over time with a very high frequency (10-30/day) and persistent in the three months before death. Many anti-seizure medications and a ketogenic diet were tried. In the three months before death the patient was taking Vigabatrin as anti-seizure medication; no other therapies were described. No changes in epilepsy features were described in the three months before death.

**Proband 6**

Proband 6 (pt.4, Nicita et al, 2015) [13] was a girl, with a *de novo* 9q33.3→9q34.12 deletion spanning about 4 Mb from 129,509,718 to 133,647,818 bp (hg19), deceased at 2 years of age for cardiopulmonary arrest. No particular events were reported at death with clinical and neurological conditions stable. Family history was negative for sudden death or other relevant comorbidities. She had severe ID and developmental delay (no developmental skills acquired, epilepsy, apostural tetraparesis, sensorineural hearing loss, left unilateral cleft lip and palate, dysmorphic facial features, large umbilical hernia, dilated renal pelvis, microcephaly, distal dystonic posturing of the lower limbs. Seizures started at 5 weeks of life with palpebral and buccal myoclonias. Ictal EEG showed epileptiform discharges in the right or left hemisphere. Focal clonic seizures, epileptic spasms, and palpebral and buccal myoclonic seizures were described over time and persistent in the three months before death. Many anti-seizure medications were tried. In the three months before death the patient was taking Zonisamide, Vigabatrin and Clobazam as anti-seizure medications without changes of drugs, but increased dosages; no other therapies were described. No changes in epilepsy features were described in the three months before death.

**Proband 10**

Proband 10 was a boy, with a *de novo* missense variant c.569G>A, p.(Arg190Gln), deceased at 7 years of age for definite SUDEP. Other genetic mutations were reported: GABRB3 c.31C>T, p.(Pro11Ser) and GRIN2A c.422C>T, p.(Thr141Met). When he passed away, an infection by COVID-19, although asymptomatic, was reported. No other events were reported at death with clinical and neurological conditions stable. Family history was negative for sudden death or other relevant comorbidities. He had mild ID, epilepsy and ataxia. Seizures started at 6 and 12 months with febrile seizures. Generalized tonic-clonic seizures and absences were described over time. In the three months before death, generalized tonic-clonic seizures with a multiple/day frequency mainly during sleep (3-6 seizures/night) were reported. An EEG performed in that period showed > 40 subclinical seizures per night. Many anti-seizure medications and a ketogenic diet were tried, and VNS was implanted. In the three months before death the patient was taking Sodium Valproate, Clobazam, Stiripentol and Cannabidiol as anti-seizure medications, and he had VNS implantation; no other therapies were described. No changes in epilepsy features were described in the three months before death.

**Proband 11**

Proband 11 was a boy, with a *de novo* missense variant c.131G>A, p.(Cys44Tyr), deceased at 8 years of age for probable SUDEP. Family history was negative for sudden death, epilepsy or other relevant comorbidities. He had moderate ID and developmental delay, epilepsy, fine motor impairment and limited social eye contact. He was able to walk without support and to say a few words. He was attending a special school with support. Seizures started at 3 months of life with episodes characterized by breath arrest and cyanosis. Focal seizures, sometimes evolving to GTC, were described over time with periods seizure-free. Seizures characterized by buccal automatisms, up rotation of the eyes and staring were reported with a weekly frequency in the three months before death. He was treated with Valproate and Levetiracetam. In the three months before death the patient was regularly taking Levetiracetam and another unspecified anti-seizure medication. No change in the treatment, and other therapies were described.

**Proband 15**

Proband 15 was a girl, with a *de novo* heterozygous missense variant c.847C>T p.(Arg292Cys), deceased at 10 years of age for definite SUDEP. No comorbidities or particular events were reported at death. Family history was negative for sudden death or other relevant comorbidities except for a first-line paternal cousin affected by epilepsy without other symptoms. She had severe ID and developmental delay, epilepsy, ataxia, cerebral palsy, and gastroesophageal reflux. She could stand without support; she could not walk. Seizures started at 18 months with tonic-clonic seizures, sometimes in clusters when the patient was sick. Generalized tonic-clonic seizures persisted over time with a multiple/week frequency in the three months before death. Many anti-seizure medications were tried over time, but they were all stopped for severe side effects (Steven-Johnson after an unspecified drug and severe constipation after all the drugs tried). She was not taking any anti-seizure medication before death, except for Clonazepam as rescue medicine. No changes in treatment and no other therapies were reported in the three months before death. No changes in epilepsy features were described in the three months before death.

**Proband 16**

Proband 16 (pt.T1915 Carvill et al, 2014) [14] was a boy, with a *de novo STXBP1* missense variant c.847G>A, p.(Glu283Lys) in the setting of Dravet syndrome, who died at 11 years of age with probable SUDEP. The events surrounding his death were not known. Another variant (*SCN9A* c.3734A>G, p.(Asn1245Ser) polymorphism) in this patient was a common benign variant. Family history was positive for depression and obsessive-compulsive disorder. He had epilepsy and severe ID with regression at age 3 years in toilet training and his language declined from 40 to 5 single words. He walked independently and had autistic features; he had viral encephalitis at 8 years of age with renal problems. Seizure onset occurred at 11 months with a cluster of afebrile generalized tonic-clonic seizures associated with a respiratory tract infection. He developed focal impaired awareness, myoclonic, atonic and tonic seizures. Seizures were sometimes associated with fever early in life. He had status epilepticus at age 10 years. In the three months prior to his death, he continued to have multiple nocturnal tonic seizures per day. Many anti-seizure medications and the ketogenic diet were tried; however, changes in the three months prior to his death were unknown.

**Proband 19**

Proband 19 was a girl, with a *de novo* large deletion including *STXBP1* gene, *ING* gene and 50 other genes, deceased at 13 years of age. The cause of death was a possible SUDEP. No particular events at death were reported except for the reappearance of seizures the day before death (the patient was seizure-free since the age of 8 months) and poor sleep in the days before death. Family history was negative for epilepsy, sudden death, or other relevant comorbidities. She had ID, developmental delay (unspecified entity), epilepsy and neurogenic bladder. She was non verbal and she was using communication devices. She was able to walk with support, but she needed a wheelchair for transports or long walks. Seizures started at 3 months of life with spasms, treated with ACTH. Between 3- and 8- months myoclonic seizures appeared. She took anti-seizure medications: ACTH from 3 to 8 months, Zonisamide from 8 months to 2 years and Lamotrigine from 2 to 13 years. She was seizure-free from the age of 8 months to 13 years. The day before death, at 13 years, she had a seizure (the mother found her in the toilet on the floor without consciousness) and the day of death she had another seizure (the mother found her unconscious on the floor, with bladder and sphincteric release). The duration of the last two seizures was around 2 minutes. Both the seizures happened when she woke up in the morning. Sleep deprivation, due to many awakenings, was described in the two days before death. For sleep disturbances, she was taking Melatonin and another unspecified medicine as rescue medication. The day before death, the patient was evaluated in the hospital for the seizure. No emergency treatment and no change in anti-epileptic therapy occurred (the patient was regularly taking Lamotrigine as anti-seizure medication). In the three months before death it was changed the medication for neurogenic bladder. Neurological and clinical conditions, except for the seizures and sleep disturbances described above, were reported as stable in the three months before death.

**Proband 20**

Proband 20 was a boy, with a *de novo* deletion del(9) (q34.11), dup(19) (q13.32), deceased at 13 years of age for subcontinuing seizures. No particular events were reported at death with clinical and neurological conditions stable, except for worsening of seizures. Family history was negative for sudden death or epilepsy. He had severe ID and developmental delay (he was not able to walk neither to speak), epilepsy, spasticity, hypotonia, tremor, cortical visual impairment. Seizures started at 1 week of life with spasms. Absences, atonic, tonic, focal, GTC seizures were described over time with a very high frequency (multiple/day) and persistent in the three months before death. Many anti-seizure medications and ketogenic diet were tried. In the three months before death he was taking Vigabatrin, Clobazam, Cannabidiol as anti-seizure medications without changes of drugs and he was also on treatment with VNS. No other therapies, except for multivitamins, were described. In the three months before death a worsening of seizures frequency until subcontinuing seizures at death was described.

**Proband 23**

Proband 23 was a boy, with a *de novo* unspecified pathogenic *STXBP1* variant, deceased at 14 years of age. The cause of death was reported as general global clinical compromise. No particular events at death were reported except for global deterioration worsened during the last months of life (severe constipation, no appetite, very low body weight, extremely low muscular strength). Family history was negative for epilepsy, sudden death, or other relevant comorbidities. He had severe ID and developmental delay (sitting at 2 years and standing only transitory at 8 years, epilepsy, hypotonia, constipation and weakness (after puberty). He was non-verbal, not able to communicate and not ambulant. His behavior changed after puberty (happy before puberty and unhappy after puberty). Seizures started at 2 weeks of life with GTC seizures and they remained the unique seizure type for the first month of life. From 2 to 6 months spasms appeared, treated with ACTH (no effect), Vigabatrin (only transitory effect), and Levetiracetam (good control on spasms from 6 months of age). From 6 months to 4 years he was seizure-free. From 4 years tonic seizures appeared with a frequency of multiple (up to 40) seizures/day. He tried many unspecified anti-seizure medications without effect until he started ketogenic diet that completely controlled tonic seizures after 6 months. From 4-5 to 11 years he was seizure-free on ketogenic diet (without any anti-seizure medication). From 11 years GTC seizures reappeared with a frequency of 2-3/day and a duration of 20 seconds, sometimes in clusters treated with rescue medicine. He continued ketogenic diet for some time, he tried again Levetiracetam and he tried other anti-seizure medications, such as Clobazam and Cannabidiol, with partial effect (slight reduction of the seizures), without complete control of the seizures. In the last three months of life he presented with 2-3 GTC seizures/day, until the last week of life, when no seizures were observed. Seizures occurred prevalently during wakefulness. He was regularly taking anti-seizure medications (Cannabidiol and Clobazam) except for the last two weeks of life, when his clinical conditions deteriorated so much that he refused food and everything else. He was also taking unspecified mio-relaxant therapy, also stopped in the last two weeks of life. No changes in epilepsy features were described in the three months before death. In the last weeks of life clinical and neurological conditions progressively deteriorated.

**Proband 24**

Proband 24 was a boy, with a *de novo* missense variant c.1651C>T p.(Arg551Cys), deceased at 15 years of age for an out of hospital cardiac arrest due to hypoxia during an epileptic seizure. No comorbidities or particular events were reported at death. Family history was negative for sudden death or other relevant comorbidities, except for two miscarriages. He had severe ID and developmental delay (before epilepsy onset (11months) he could pull himself up to a standing position; after epilepsy onset he presented with severe developmental delay with slow progression and periods of stagnation. He started to walk at 2-4 years of age, but this ability regressed over time). No speech, epilepsy, autism spectrum disorder, minimal eye contact, abnormal muscular tone (hypotonia when younger and legs spasticity when older) and tube feeding were reported. Seizures started at 11 months with a seizure described as change of color of the face and shaking of all the body, followed the day after by epileptic status reported as focal epileptic status with impaired awareness. Epilepsy was reported as drug-resistant and characterized by generalized and focal seizures. At the age of 13 years, the patient presented with generalized tonic-clonic, focal seizures, apneas, mostly during the night, and episodes of reduced consciousness diagnosed as non-convulsive seizures. Many hospitalizations for frequent seizures were reported (20 at the age of 8 years with status epilepticus followed by Todd’s palsy at 5 years). Many anti-seizure medications were tried; Vagus nerve stimulation was also tried, but stopped for swallowing disturbances. Chloralhydrate was used for restlessness at night (at 3 years, not effective). One year before death his clinical conditions looked stable with better control of seizures on treatment with Brivaracetam (reduced seizure frequency during the day) and improvement of sleeping and eating. Two months before his death he had an infection with high fever and drowsiness, recovered after antibiotic treatment.

**Proband 27**

Proband 27 was a girl, with a *de novo* truncating variant c.1162C>T, p.(Arg388P*), deceased at 16 years of age for possible SUDEP. No particular events at death were reported. Family history was negative for epilepsy, sudden death, or other relevant comorbidities. She had severe ID and developmental delay (holding the neck gained at 9 months, sitting gained at 1 year with balance problems, standing gained at 2 years and walking gained at 2.5 years). She could say a couple of words (mum and dad) and she was able to express what she wanted (for example if she wanted something to eat), she was social, with good sense of humor. She could walk without support. Epilepsy, tremor, impaired fine motor skills, balance instability were also reported. At 8-10 years of age the patient experienced sleep disturbances and need a temporary treatment with an unspecified anti-depressive medication for sleep with good effect, stopped after three weeks. Seizures started at 15 years of age (three weeks before death) with a tonic seizure (deviation of the eyes and of the head to the left, rigidity of the body and cyanosis) of 3-4 minutes. The patient was hospitalized and she presented with 2 other tonic seizures, with the same characteristics, but shorter (1-2 min) the same day at the hospital. No specific treatment was started. Seizures happened during wakefulness. After two days of observation in the hospital, she was sent home with the prescription of endo-rectal Diazepam as rescue medicine. No more seizures were observed after and no anti-seizure medications were used. Neurological and clinical conditions, except for the onset of epilepsy three weeks before death, were reported as stable in the three months before death.

**Proband 29**

Proband 29 was a girl, with a *de novo* deletion in the region 9q44 (including the *STXBP1* gene), deceased at 18 years of age for global clinical compromise. No comorbidities or relevant events were reported when she passed away. Her family history was negative for sudden death or relevant morbidities, except for a maternal aunt affected by Turner syndrome. She had severe ID and motor and language delay with regression in childhood and at puberty (she could walk without support at 5 years of age, and regressed the ability to walk at puberty, when she became wheelchair-bound. She spoke few words in infancy, but lost this ability at 7 years; from this age she could only say yes and no). Epilepsy, hypotonia, ataxia, neurological bladder and constipation were also reported. At puberty, she developed lethargy, loss of muscle strength and loss of all the skills. Seizures started at birth with buccal automatism followed by respiratory arrest. She developed status epilepticus at 6 days of life. She was diagnosed with “Ohtahara-like” syndrome and started a treatment with Phenobarbital with good seizure control for 11 months. At 2-3 years of age she became severely hyperactive, she didn’t sleep and her seizure frequency increased. At 3 years of age, atonic drops appeared; another treatment was started with complete control of the seizures for 1 year. For sleep problems, she started Clonidine, that she continued until 15 years of age, when she became lethargic. At 4 years of age clonic and generalized tonic-clonic (GTC) seizures appeared. She tried many anti-seizure medications and ketogenic diet over time, without control of seizures. In the three months before death, she presented with multiple focal seizures/day (up to 100 seizures/day of few duration). She was taking Zonisamide, Cannabidiol, Phenitoin, and Clobazam, as anti-seizure medications at death. By the end of her life she was very lethargic (she was sleeping for 4 days and waking up only for 45 minutes). No changes in epilepsy features, neither in treatments were described in the three months before death.

**Proband 30**

Proband 30 was a girl, with a *de novo* missense variant c.1651C>T, p.(Arg551Cys), deceased at 19 years of age for choking after a seizure. No particular events at death were reported. Another genetic variant (*SCN9A* c.684C>G, p.(Ile228Met), likely pathogenic) was revealed and reported in literature in association with Dravet syndrome, axonal neuropathy, small fiber neuropathy, and erythromelalgia. Family history was negative for epilepsy, sudden death, or other relevant comorbidities. She had severe ID and developmental delay (walking gained at 2.5 years of age). She was non-verbal (she never babbled or spoke, at 2 years she was able to produce some sounds, but she lost this ability). She could walk without support. Epilepsy, hypotonia, constipation and gastroesophageal reflux were also reported. Seizures started at 9 months of life with focal tonic seizures. At 11 months of life spasms appeared with a pattern of burst suppression at EEG, and were treated with ACTH with good effect, stopped after 1 month. Focal tonic seizures continued over time, sometimes evolving to extension to generalized tonic clonic seizure. When she was younger (less than 10 years of age) seizures presented in clusters with a frequency of multiple (20-30) seizures/day. The frequency of seizures decreased when the patient was older than 10 years of age (1-2 seizures/week) with worsening during menstruations. EEG showed bilateral interictal epileptiform discharges. Seizures were never completely controlled by anti-seizure medications. She tried over time many anti-seizure medications. In the three months before death she was taking Levetiracetam and Lamotrigine as anti-seizure medications and she had focal tonic seizures, with a frequency of 1-2 seizures/week, sometimes evolving to extension to generalized tonic clonic seizure (2-3 times/month). No change in anti-epileptic treatment or epilepsy features were reported in the three months before death. Sleep disturbances, consisting in awakenings during the night, were described, but well treated with Melatonin and Clonidine. She was also taking Ranitidine for gastroesophageal reflux and anti-histaminic medicine for seasonal allergies, none of them changed in the three months before death. Neurological and clinical conditions were reported as stable in the three months before death.

**Proband 31**

Proband 31 was a girl, with a *de novo* unspecified pathogenic *STXBP1* variant, deceased at 19 years of age due to progressive deterioration. No particular events at death were reported, except for a deterioration of the general and neurological conditions was described (lethargy, worsening of the seizures and rigidity). Family history was negative for epilepsy, sudden death, or other relevant comorbidities. She had severe ID and developmental delay (she could stand, she was not able to walk). She was non-verbal and not ambulant. Epilepsy, tremor, rigidity, neurogenic bladder, instability were also reported. Seizures started at 5 days of life with a GTC seizure. GTC seizures continued over time with a frequency of multiple seizures (1-30)/day from 5 days to 4 years, seizure-free period for 1-2 years at 4-5 years of age (she continued anti-seizure medications because EEG showed IEDs), multiple seizures/week (1-2/week) from 6 to 11 years (changed anti-seizure medications), multiple seizures/day from 11 to 19 years with periods seizure-free (the longest months). At 7 years a worsening of general and neurological conditions was observed (she lost the ability to stand and to eat something on her own, she lost weight, she presented with decreased muscular strength and increased tremor, she was less interactive). Seizures characteristics and frequency didn’t change. She was taking as anti-seizure medication Valproate. An MRI performed at 9 years of age showed atrophy. Valproate was stopped at 9 years and Levetiracetam was started. From 9 to 11 years an improvement of general and neurological conditions was observed (she didn’t completely recover as before, but she was more interactive, she gained weight, she presented with more strength and less tremor, and she regained the ability to stand and eat something on her own). At 11 years puberty arrived and seizures worsened as described above. At the age of 14 years absences also appeared and they continued over time with a frequency of multiple seizures/day with periods seizure-free (the longest months). At 14-15 years of age seizures presented in clusters and she presented periods of developmental regression and regain with never completely regain, she started to be more sleepy and less happy. From 17 to 19 years: the seizure-free periods decreased and were not longer than one week. Seizures have always worsened during the menstruation periods. At 17-19 years of age, a general deterioration was observed (she became more lethargic (she was sleeping 20 hours/day) and isolated in her world). Many anti-seizure medications and VNS were tried over time without control of the seizures. In the last three months of life, no seizure was observed, and no change in anti-epileptic treatment or other medicines was reported. At death she was taking Lamotrigine, Levetiracetam and two more unspecified anti-seizure medications, Fluoxetine for episodes of “stopping the breathing” (the episodes stopped with Fluoxetine) and oral contraceptives.

**Proband 35**

Proband 35 (pt.3, Stamberger et al, 2022) [4] was a boy, with a *de novo* missense variant c.701AYG, p.(D234G), deceased at 36 years of age for complications after surgery (indication of surgery and exact cause of death are unknown). Unknown if particular events occurred at death. Family history was negative for epilepsy or other relevant comorbidities. He had severe ID and he was non-verbal. Serious complex behavioral disorder (agitation and aggressive behavior) was described in childhood. Epilepsy, mild ataxia and mild coordination impairment were also reported. Seizures started at 10 months of life. The patient presented with GTC seizures and focal seizures with impaired awareness (tonic and not motor). Epilepsy was described as drug-resistant. Many anti-seizure medications have been tried over time. Epilepsy features, anti-seizure medications, eventual other medicines, in the three months before death are unknown.
